# Automatic assessment of disproportionately enlarged subarachnoid-space hydrocephalus from 3D MRI using two deep learning models

**DOI:** 10.3389/fnagi.2024.1362637

**Published:** 2024-03-15

**Authors:** Shigeki Yamada, Hirotaka Ito, Hironori Matsumasa, Satoshi Ii, Tomohiro Otani, Motoki Tanikawa, Chifumi Iseki, Yoshiyuki Watanabe, Shigeo Wada, Marie Oshima, Mitsuhito Mase

**Affiliations:** ^1^Department of Neurosurgery, Nagoya City University Graduate School of Medical Science, Nagoya, Japan; ^2^Interfaculty Initiative in Information Studies/Institute of Industrial Science, The University of Tokyo, Tokyo, Japan; ^3^Medical System Research & Development Center, FUJIFILM Corporation, Tokyo, Japan; ^4^Faculty of System Design, Tokyo Metropolitan University, Tokyo, Japan; ^5^Department of Mechanical Science and Bioengineering, Graduate School of Engineering Science, Osaka University, Osaka, Japan; ^6^Department of Behavioral Neurology and Cognitive Neuroscience, Tohoku University Graduate School of Medicine, Sendai, Japan; ^7^Division of Neurology and Clinical Neuroscience, Department of Internal Medicine III, Yamagata University School of Medicine, Yamagata, Japan; ^8^Department of Radiology, Shiga University of Medical Science, Otsu, Japan

**Keywords:** artificial intelligence, deep learning, MRI, disproportionately enlarged subarachnoid-space hydrocephalus, idiopathic normal pressure hydrocephalus, chronic hydrocephalus in adults, hakim disease, tightened sulci in high convexities

## Abstract

**Background:**

Disproportionately enlarged subarachnoid-space hydrocephalus (DESH) is a key feature for Hakim disease (idiopathic normal pressure hydrocephalus: iNPH), but subjectively evaluated. To develop automatic quantitative assessment of DESH with automatic segmentation using combined deep learning models.

**Methods:**

This study included 180 participants (42 Hakim patients, 138 healthy volunteers; 78 males, 102 females). Overall, 159 three-dimensional (3D) T1-weighted and 180 T2-weighted MRIs were included. As a semantic segmentation, 3D MRIs were automatically segmented in the total ventricles, total subarachnoid space (SAS), high-convexity SAS, and Sylvian fissure and basal cistern on the 3D U-Net model. As an image classification, DESH, ventricular dilatation (VD), tightened sulci in the high convexities (THC), and Sylvian fissure dilatation (SFD) were automatically assessed on the multimodal convolutional neural network (CNN) model. For both deep learning models, 110 T1- and 130 T2-weighted MRIs were used for training, 30 T1- and 30 T2-weighted MRIs for internal validation, and the remaining 19 T1- and 20 T2-weighted MRIs for external validation. Dice score was calculated as (overlapping area) × 2/total area.

**Results:**

Automatic region extraction from 3D T1- and T2-weighted MRI was accurate for the total ventricles (mean Dice scores: 0.85 and 0.83), Sylvian fissure and basal cistern (0.70 and 0.69), and high-convexity SAS (0.68 and 0.60), respectively. Automatic determination of DESH, VD, THC, and SFD from the segmented regions on the multimodal CNN model was sufficiently reliable; all of the mean softmax probability scores were exceeded by 0.95. All of the areas under the receiver-operating characteristic curves of the DESH, Venthi, and Sylhi indexes calculated by the segmented regions for detecting DESH were exceeded by 0.97.

**Conclusion:**

Using 3D U-Net and a multimodal CNN, DESH was automatically detected with automatically segmented regions from 3D MRIs. Our developed diagnostic support tool can improve the precision of Hakim disease (iNPH) diagnosis.

## 1 Introduction

Chronic hydrocephalus in adults is called “normal-pressure hydrocephalus (NPH)” because of the absence of intracranial hypertension symptoms, and has been largely classified into idiopathic NPH (iNPH) or secondary NPH (sNPH), which develops after subarachnoid hemorrhage, trauma or infection by Adams et al. (1965). Since international and Japanese guidelines for the management of iNPH were published and revised (Ishikawa and Guideline Committe for Idiopathic Normal Pressure Hydrocephalus, Japanese Society of Normal Pressure Hydrocephalus, 2004; Marmarou et al., 2005; Mori et al., 2012; Nakajima et al., 2021), there has been an increased focus on iNPH, which is known to present with a triad of symptoms: gait disturbance, cognitive dysfunction, and incontinence. Recently, an international collaborative group examining the contemporary classifications, terminology, and definitions of chronic hydrocephalus in adults proposed renaming iNPH to “Hakim disease” (Tullberg et al., 2024), because many experts questioned the term iNPH, i.e., “normal pressure” indicates normal intracranial pressure and “idiopathic” implies unknown causes. If this condition is left untreated, symptoms gradually progress with a corresponding decrease in independence (Yamada et al., 2017c, 2021a), eventually leading to death (Andren et al., 2020, 2021). Recently, Hakim disease (iNPH) has been recognized as a common disease among the elderly, with a large proportion of Hakim patients potentially present in a superaged society. Based on previous epidemiological studies (Iseki et al., 2009, 2022; Jaraj et al., 2014; Kuriyama et al., 2017; Constantinescu et al., 2023), however, the probability of Hakim patients receiving appropriate treatment is estimated to be less than 10% of all potential patients, and there are large regional differences. Since Hakim disease is still undetected or misdiagnosed in many countries, an easier and more reliable method to identify Hakim disease is desperately needed. The main reason for missed detection or misdiagnosis of Hakim disease, even when advanced imaging technologies are widely available, is that Hakim disease is often less prominent with ventricular dilatation (VD) and more prominent with Sylvian fissure dilation (SFD), which is also caused by medial temporal lobe atrophy, a well-known imaging feature specific to Alzheimer’s disease and mild cognitive impairment (Coupe et al., 2019; Wang et al., 2022). Consequently, VD and SFD are easily misinterpreted as brain atrophy related to neurodegenerative diseases including Alzheimer’s disease (McCarty et al., 2019; Virhammar et al., 2021). To distinguish Hakim disease from focal cerebral atrophy, disproportionately enlarged subarachnoid space hydrocephalus (DESH) (Hashimoto et al., 2010; Shinoda et al., 2017; Gunter et al., 2019; McCarty et al., 2019; Virhammar et al., 2021), including tightened sulci in the high convexities (THC) (Sasaki et al., 2008; Ishikawa et al., 2010; Narita et al., 2016; Yamada et al., 2016a, 2021b, 2023a; Yamada and Mase, 2023), have recently been noted as the most important imaging features specific to Hakim disease. DESH refers to unbalanced CSF distribution in the subarachnoid space (SAS), i.e., simultaneous occurrence of SFD and THC. Although DESH is increasingly recognized as a neuroimaging hallmark of Hakim disease, subjective evaluation of DESH remains ambiguous and often confusing, with judgments differing among experts (Sasaki et al., 2008; Ishikawa et al., 2010; Narita et al., 2016; Shinoda et al., 2017). Therefore, we aimed (a) to develop artificial intelligence (AI) to automatically and accurately extract volumes of interest (VOIs) from 3D T1-weighted or T2-weighted MRIs in Hakim patients and healthy subjects at an accuracy near, equal to or greater than that of expert evaluators, (b) to develop AI to automatically detect DESH as well as VD, SFD, and THC from VOIs, and (c) to establish that the newly defined indices related to DESH could accurately determine DESH.

## 2 Materials and methods

### 2.1 Study population

From our previous study using 3D T2-weighted MRI data acquired on MAGNETOM Skyra (Siemens AG, Munich, Germany) until September 2019 (Yamada et al., 2015, 2016a,b, 2017b, 2019), 14 patients (10 Hakim patients and 4 volunteers) were included in this study. Subsequently, from our recent study (Yamada et al., 2020, 2021c, 2023a,b,c), 115 patients (26 Hakim patients and 89 volunteers) who had undergone 3D T1-weighted and T2-weighted MRIs on a Discovery MR 750 W (GE Healthcare, Milwaukee, Wisconsin, United States) from October 2019 to January 2022, and 51 participants (6 Hakim patients and 45 volunteers) on a Signa Architect 3.0 T (GE Healthcare) from February 2022 to May 2022 were enrolled in this study. Healthy volunteers aged ≥20 years, were recruited from among medical staff, students, and their family members by open recruitment. The inclusion criteria for this study were as follows: individuals with no previous history of brain injury, brain tumor, or cerebrovascular disease on brain MRI examinations, and individuals who had never undergone brain CT or MRI and had no neurological symptoms, including compromised cognitive function. Three volunteers were incidentally detected with small unruptured intracranial aneurysms, but they were included in this study because small unruptured intracranial aneurysms were unlikely to affect brain and CSF volumes. One examination of 3D T1-weighted MRI in a healthy volunteer was excluded, because the MRI sequence and orientation differed completely from those of other images. Among 138 healthy volunteers, one was judged to have DESH, VD, and THC but not SFD, and was diagnosed with asymptomatic ventriculomegaly with features of iNPH on MRI (AVIM) (Iseki et al., 2009). All patients were diagnosed with or without Hakim disease, according to the third edition of the Japanese guidelines for management of iNPH (Nakajima et al., 2021). Among the 42 Hakim patients, 40 had triad symptoms of gait disturbance, cognitive impairment, and urinary incontinence, whereas two had very mild or no objective symptoms and did not undergo a CSF tap test or shunt surgery, and therefore would be classified as having AVIM. Overall, 18 patients (42%) underwent the CSF tap test, 21 patients (50%) underwent CSF shunt surgery, and their symptoms improved by ≥1 point on the modified Rankin Scale and/or the Japanese grading scale (Nakajima et al., 2021). Finally, 138 volunteers and 42 patients diagnosed with Hakim disease were included in this study (Table 1).

**Table 1:** Clinical characteristics of the study population.

	Total	Skyra	MR 750 W	Architect	
		Siemens	GE	GE	*P*
	180	14	115	51	
Hakim disease: volunteer	42: 138	10: 4	26: 89	6: 45	<0.001
Male: female	78: 102	10: 4	48: 67	20: 31	0.0887
Mean age (years)	55.2 ± 19.7	74.3 ± 9.7	49.3 ± 18.8	63.2 ± 17.7	<0.001
≦50 years	97	1	82	14	<0.001
60 years	28	0	13	15	
≧70 years	55	13	20	22	
DESH	43	10	26	7	<0.001
VD	45	10	25	10	<0.001
THC	42	10	25	7	<0.001
SFD	32	9	17	6	<0.001
AD	4	1	2	1	0.3413

P; probability values of the proportions among MRI machines based on the Chi-square test, and *p* value of the mean age were calculated by the Kruskal-Wallis test. DESH; disproportionately enlarged subarachnoid-space hydrocephalus, VD; ventricular dilatation, THC; tightened sulci in the high-convexity, SFD; Sylvian fissure dilation.

### 2.2 Ethics approval

This study was approved by the ethics committees for human research at our institutes (IRB Number: 60-22-0083, R2019-227). Healthy volunteers provided written informed consent and underwent MRI examinations, after explaining the aim of this study and the potential for detection of diseases in the brain. Patients’ MRI data were obtained in an opt-out method, after their personal information was anonymized in a linkable manner.

### 2.3 Image acquisitions

The sequence parameters of T1-weighted 3D magnetization prepared rapid gradient echo (MPRAGE) were as follows: TR, 2471 ms; TE, 3.13 ms; inversion time, 1,000 ms; flip angle, 8°; matrix 256 × 256; voxel size, 0.9 × 0.9 × 0.9 mm; and acquisition time, approximately 4 min. The sequence parameters of 3D T2-weighted Cube were as follows: TR, 2000 ms; TE, 85.3 ms; matrix 288 × 288; voxel size, 0.8 × 0.8 × 0.8 mm; and acquisition time, approximately 4 min. The sequence parameters of 3D T2-weighted sampling perfection with application optimized contrast using the variable flip-angle evolution (SPACE) were as follows: TR, 2800 ms; TE, 286 ms; matrix 192 × 192; voxel size, 0.6 × 0.6 × 0.9 mm; and acquisition time, approximately 4 min.

### 2.4 Preparation for data processing of deep learning

**Figure 1:**
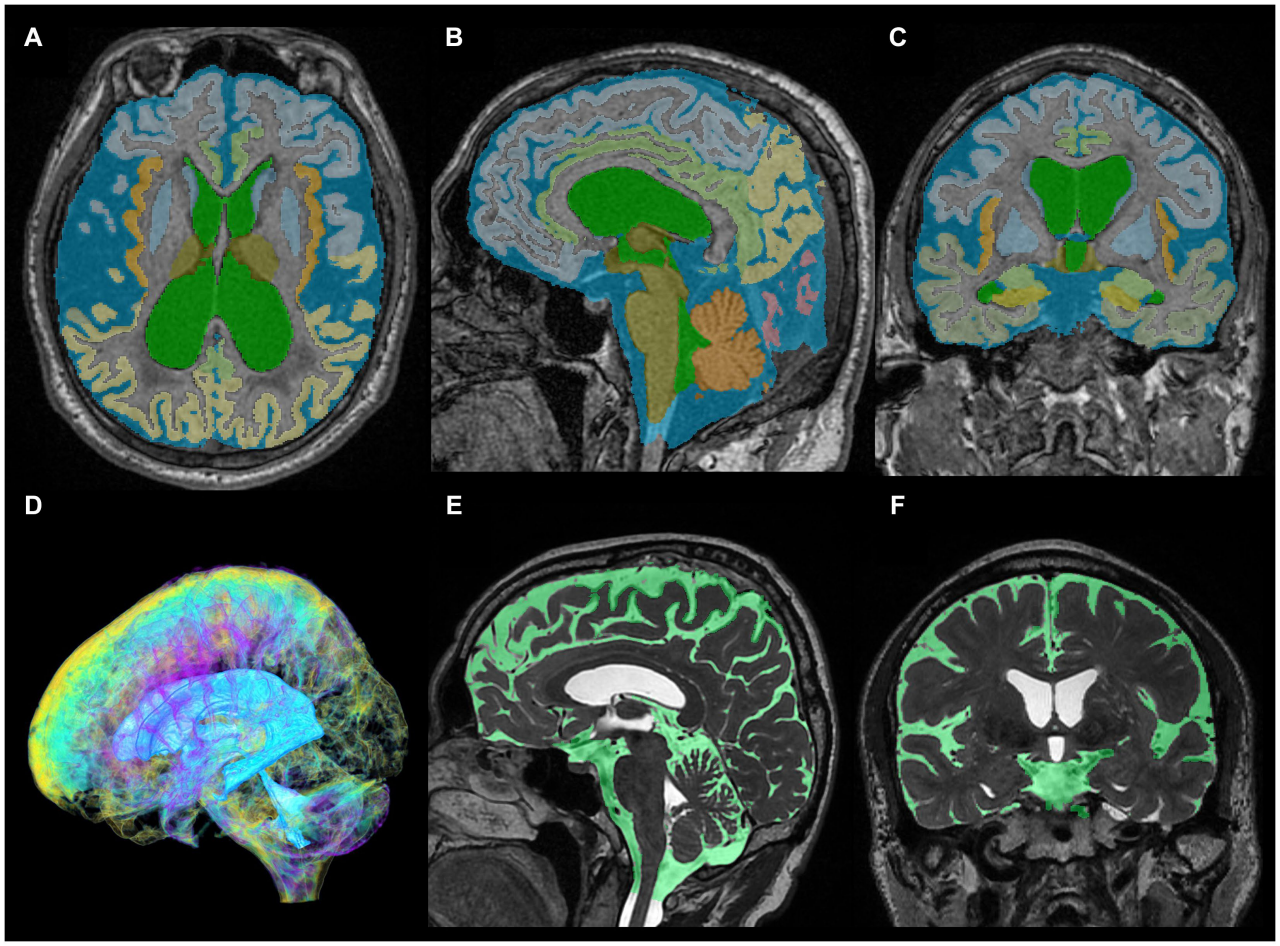
Segmentation from three-dimensional T1- and T2-weighted MRI. The upper axial **(A)**, sagittal **(B)**, and coronal **(C)** images on 3D T1-weighted MRI show the results for fully automatically segmented regions, including total ventricles (green) and total subarachnoid spaces (marine blue) of a representative patient with Hakim disease and DESH, using the Brain Subregion Analysis application on the 3D volume analyzer SYNAPSE 3D workstation (FUJIFILM Corporation). The lower three-dimensional **(D)**, sagittal **(E)**, and coronal **(F)** images on 3D T2-weighted MRI show the results of manually segmented total ventricles (light blue in **D**) and total subarachnoid spaces (light green in **E,F**) of a representative healthy elderly volunteer.

**Figure 2:**
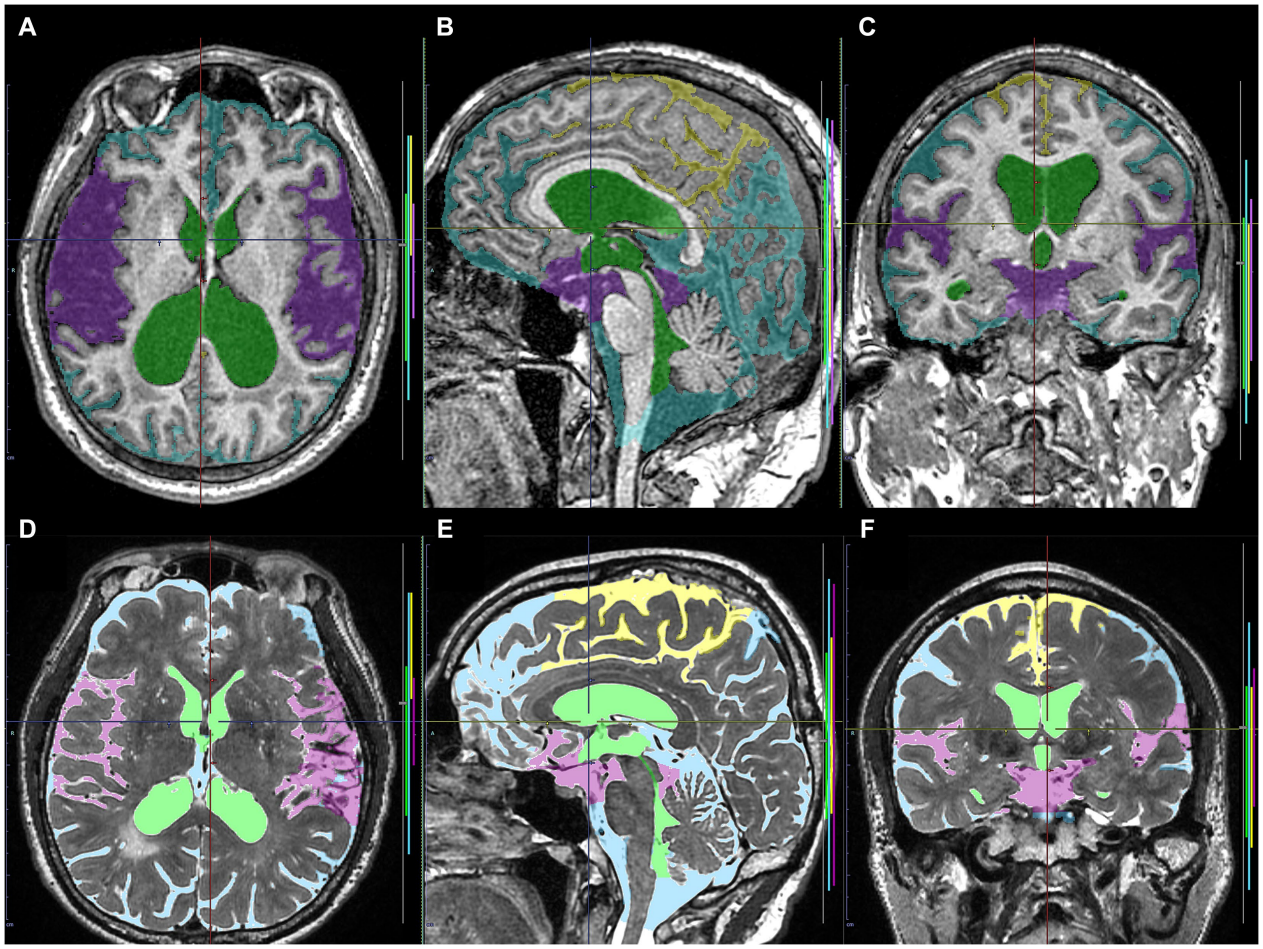
Input image masks as the ground truth labels transferred to the cloud-based AI development service. The upper axial **(A)**, sagittal **(B)**, and coronal **(C)** images on 3D T1-weighted MRI in the same Hakim patient as the upper panel of Figure 1 show the input image masks including total ventricles (green), Sylvian fissure and basal cistern (purple), high-convexity part of the subarachnoid space (yellow), and the other subarachnoid spaces (marine blue). The lower axial **(D)**, sagittal **(E)**, and coronal **(F)** images on 3D T2-weighted MRI in the same healthy volunteer as the lower panel of Figure 1 show the input image masks for deep learning including total ventricles (light green), Sylvian fissure and basal cistern (pink), high-convexity part of the subarachnoid space (yellow), and the other subarachnoid spaces (light blue).

As ground truth labels in our AI models, input image masks for volumetric semantic segmentation on the 3D T1-weighted MRI were created by combining manual segmentation with the 3D Viewer and fully automatic segmentation with the Brain Subregion Analysis applications (Figures 1A–C) on an independent 3D volume analyzer workstation (SYNAPSE 3D; FUJIFILM Corporation, Tokyo, Japan). In the Brain Subregion Analysis application, intracranial spaces were segmented fully automatically into 26 subregions including ventricles and SAS within 1 min (Yamada et al., 2023c). The input image masks from 3D T2-weighted MRI were also created using our original method, combining a simple threshold algorithm and manual segmentation (Figures 1D–F), as previously reported (Yamada et al., 2015, 2016a,b). Total SAS were further segmented into the Sylvian fissure and basal cistern, and the high-convexity SAS, which was defined as the location above the body of the lateral ventricles, with the lateral end 3 cm from the midline, the posterior end in the bilateral posterior parts of the callosomarginal sulci, and the anterior end on the coronal plane perpendicular to the AC–PC line passing through the front edge of the genu of the corpus callosum (Figure 2; Supplementary Videos S1–S4) (Yamada et al., 2023a). All input image masks as the ground truth labels were transferred to the SYNAPSE Creative Space for cloud-based AI development service (FUJIFILM Corporation). All masks were processed and formatted into a form that could utilize the training or inference process. Regarding the output of the inference process, feature maps were obtained. Overall, 159 T1-weighted images were assigned to 110 images for training, 30 for internal and 19 for external validation (test), and 180 T2-weighted images were assigned to 130 images for training, 30 for internal validation and 20 for external validation. Inference was performed in the images for internal validation and external validation.

### 2.5 Deep learning tasks

**Figure 3:**
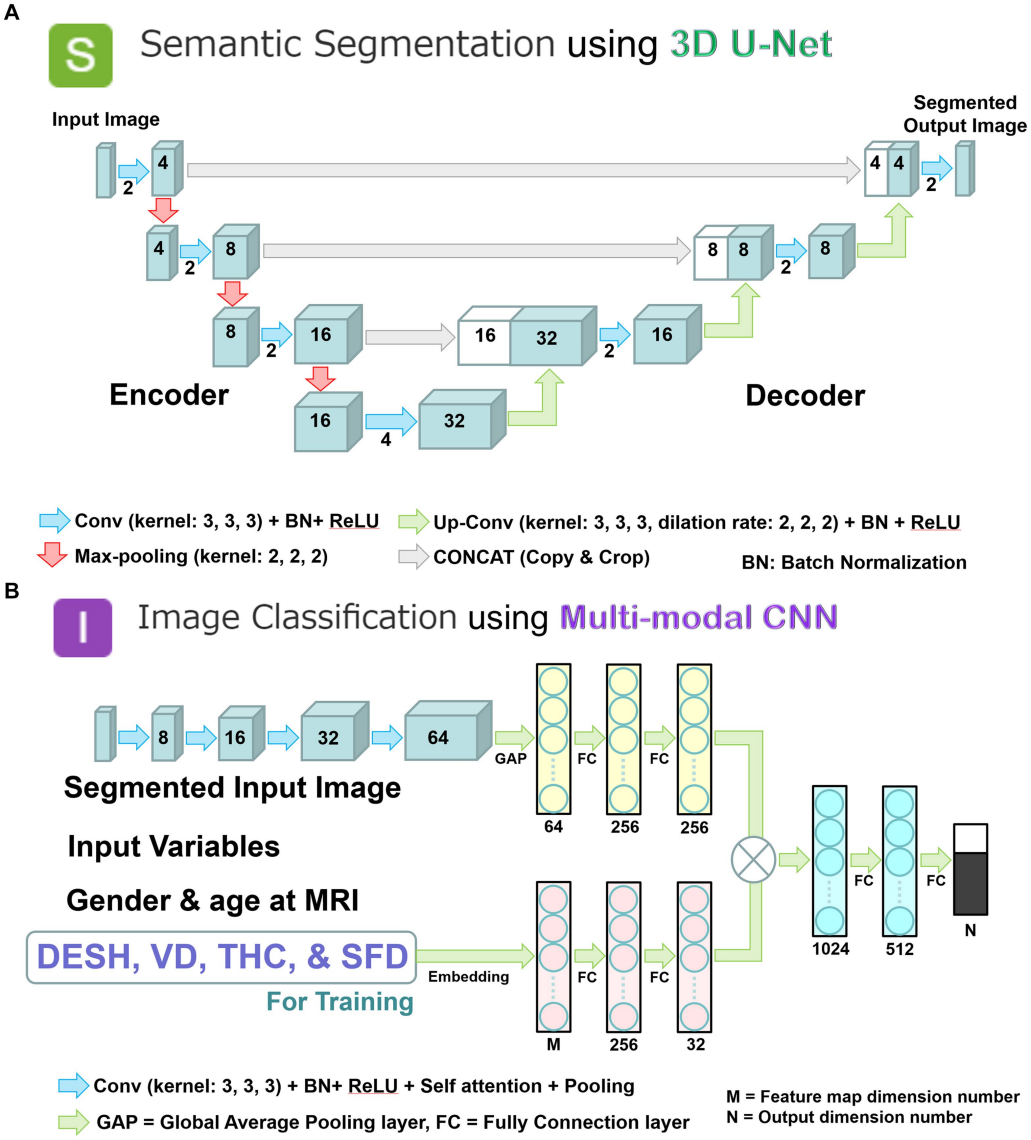
Two combined deep learning models; and multimodal convolutional neural network for image classification. **(A)** 3D U-Net model with four layers for volumetric semantic segmentation task. Each blue box corresponds to a multi-channel feature map. The number of channels is denoted on front of the box. White boxes indicate copied feature maps. The color arrows indicate each process: light blue arrows indicate convolution (Conv) with kernel size (3, 3, 3) in addition to batch normalization (BN) and rectified linear unit (ReLU) activation layer; red arrows indicate max-pooling with kernel size (2, 2, 2); green arrows indicate up-convolution (Up-Conv) with kernel size (3, 3, 3) and dilation rate (2, 2, 2) in addition to BN and ReLU; and gray arrows indicate direct concatenation from each encoding layer of feature map extracted by downsampling to the corresponding decoding layer of feature map by upsampling. Signal values were normalized by percentile (minimum 0.05, maximum 0.95) as a preprocessing step. **(B)** Multimodal convolutional neural network for image classification task. Each blue box corresponds to a multi-channel feature map with the number of channels denoted on the front of the box. The color arrows indicate each process: purple arrows indicate convolution (Conv) with kernel size (3, 3, 3) in addition to batch normalization (BN), rectified linear unit (ReLU) activation, self-attention, and pooling layer; turquoise blue arrows indicate global average pooling (GAP) or fully connection (FC) layer. In the embedding layer, all input variables were transformed into the feature maps. At the end of the last convolutional layer, the final feature maps were fed to a softmax activation function to generate a probability score for each class. The image intensities of input images were normalized to [0, 1] by their maximum and minimize values.

We combined two deep learning models to employ a two-step method of automatic detection of DESH with segmented volumes and indices. In the first step, the volumetric semantic segmentation task employed a 3D U-Net with four layers, consisting of 3D convolution with a batch normalization layer, ReLU activation layer, max pooling layer, and 3D up-convolution layer (Figure 3A). Signal values were normalized by percentile (minimum 0.05, maximum 0.95) as a preprocessing step. To compensate for voxel detail, feature maps are concatenated from each encoding layer of feature extraction by downsampling to the corresponding decoding layer of feature assignment by upsampling. In the second step, the image classification task employed a multimodal convolutional neural network (CNN) (Figure 3B). As ground truth labels for the image classification task, the presence or absence of DESH, VD, THC, and SFD was determined by a neurosurgeon and a radiologist, both experts in imaging diagnosis of Hakim disease, through consensus reading. Input data included the presence of DESH, VD, THC, and SFD, in addition to age at MRI, gender, and the same image masks used in the first step volumetric semantic segmentation task (Figure 3B). For the output of the image classification task, the intracranial CSF space mask was used to determine the presence or absence of DESH, and the masks for the total ventricle, high-convexity SAS, and Sylvian fissure and basal cistern were used to determine the presence of VD, THC, and SFD, respectively. In the embedding layer, all input variables were transformed into feature maps. At the end of the last convolutional layer, the final feature maps were fed to a softmax activation function to generate a probability score for each class. Image intensities of input images were normalized to [0, 1] by their maximum and minimum values. Augmentations including rotation, scaling, and translation of the input image masks were made to improve generalizability and accuracy in the semantic segmentation and image classification tasks. The generalizability of these augmentations would help reduce effects from differences between manufacturers, imaging protocols or individuals, and increase the robustness of our AI model.

### 2.6 Three-dimensional volumetric index

The “DESH index” was defined as the combined volume of total ventricles and Sylvian fissure and basal cistern divided by the high-convexity SAS volume. As supplemental indices for DESH, the “Venthi index” was defined as the total ventricular volume divided by the high-convexity SAS volume, and the “Sylhi index” was defined as the volume of the Sylvian fissure and basal cistern divided by the high-convexity SAS volume. These three indices were calculated by the manually and automatically segmented volumes.

### 2.7 Statistical analysis

Mean age and segmented volumes were compared using the Mann–Whitney-Wilcoxon test. The chi-square test was used to compare the proportions between Hakim patients and healthy volunteers. To quantify the performance, e.g., the accuracy of the volumetric semantic segmentation, the Dice coefficient score for the loss function was calculated as 2 * |X ∩ Y| + epsilon(1e-4)/(|X| + |Y| + epsilon(1e-4)) in the validation study. X and Y were the prediction and correct, binary [0, 1] output per voxel. The relationships between the manually and automatically segmented volumes were also examined using Pearson’s correlation coefficient (r) and 95% confidential intervals (CIs). For the image classification task, the accuracy and softmax probability score for the detection of DESH, VD, THC, and SFD were analyzed. The area under the receiver-operating characteristic curves (AUCs) and optimal thresholds with 95% CIs for detecting DESH were calculated to maximize the sum of the sensitivities and specificities. All missing variables were considered deficit data, and no other variables were adjusted. A probability value (P) of <0.001 was considered to be statistically significant. R software (version 4.2.1, R Foundation for Statistical Computing, Vienna, Austria, http://www.R-project.org) was used for all statistical analyses.

## 3 Results

### 3.1 Dataset for deep learning models

We prepared 180 datasets of 3D T2-weighted MRIs and 159 datasets of 3D T1-weighted MRIs. All 3D T1-weighted MRIs were MPRAGE sequence, and 166 3D T2-weighted MRIs were Cube sequence and 14 were SPACE sequence. For both deep learning models, 110 T1- and 130 T2-weighted MRIs were used for training, 30 T1- and 30 T2-weighted MRIs for internal validation, and the remaining 19 T1- and 20 T2-weighted MRIs for external validation. The allocation of the number of DESH or non-DESH is shown in Table 2.

**Table 2:** Assignment of MRI datasets for deep learning.

	3D T1-WI MRI			3D T2-WI MRI			
	Total	MR 750 W	Architect	Total	Skyra	MR 750 W	Architect
Training	110	72	38	130	8	77	45
(DESH: non-DESH)	15: 95	12: 60	3: 35	28: 102	5: 3	16: 61	7: 38
Internal validation	30	20	10	30	4	20	6
(DESH: non-DESH)	8: 22	5: 15	3: 7	10: 20	3: 1	7: 13	0: 6
External validation (test)	19	16	3	20	2	18	0
(DESH: non-DESH)	4: 15	3: 13	1: 2	5: 15	2: 0	3: 15	0

DESH; disproportionately enlarged subarachnoid-space hydrocephalus. Non-DESH; absence of DESH.

### 3.2 Volumetric semantic segmentation

**Figure 4:**
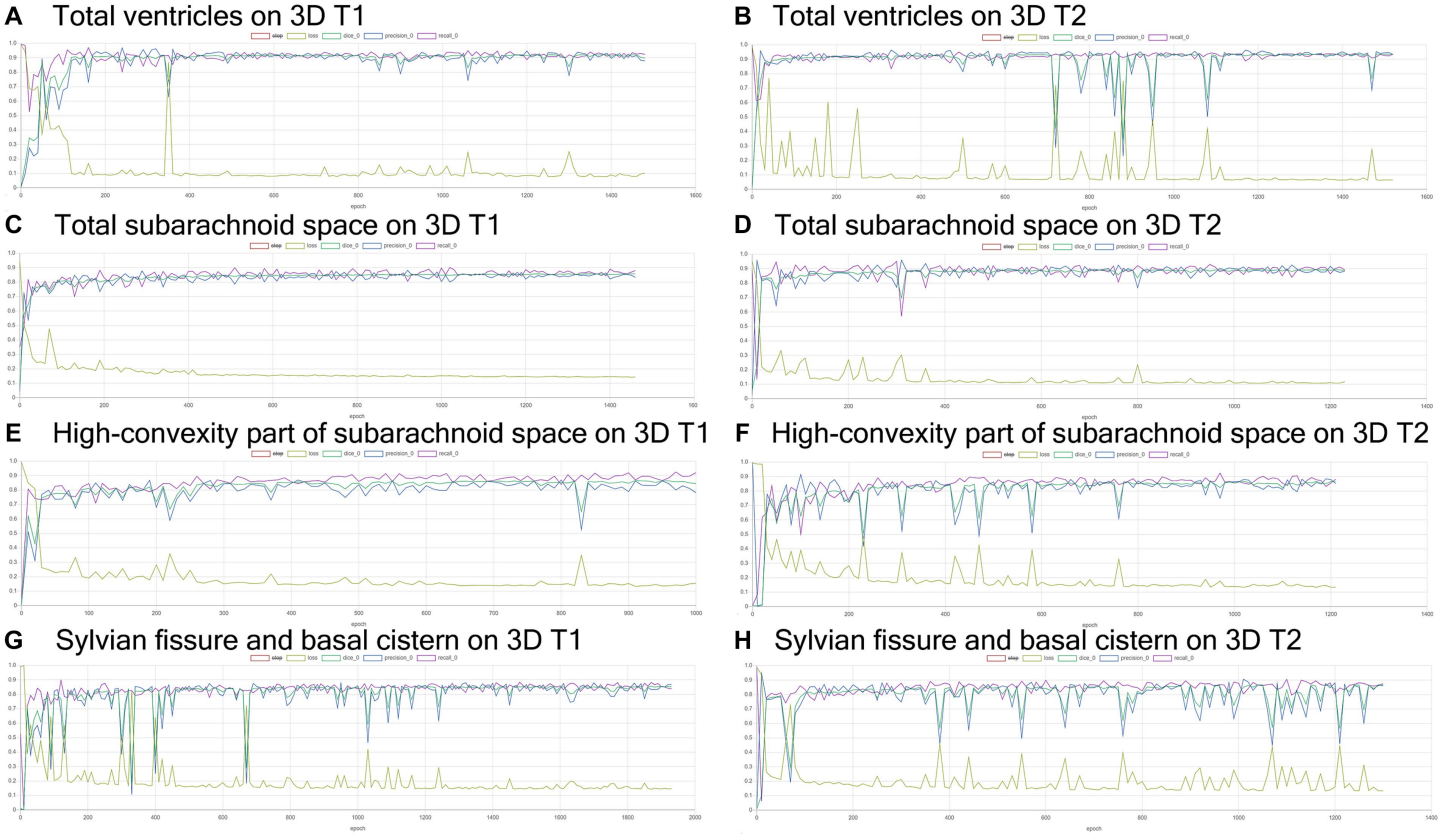
Results of training for deep learning of the semantic segmentation. The Dice scores (emerald green line), loss (lime green line), precision (blue), and recall (purple) for the automatically segmented volumes of the total ventricles **(A,B)**, total subarachnoid spaces **(C,D)**, high-convexity part of the subarachnoid space **(E,F)**, and Sylvian fissure and basal cistern **(G,H)** on 3D T1-weighted **(A,C,E,G)** and T2-weighted MRIs **(B,D,F,H)**.

**Figure 5:**
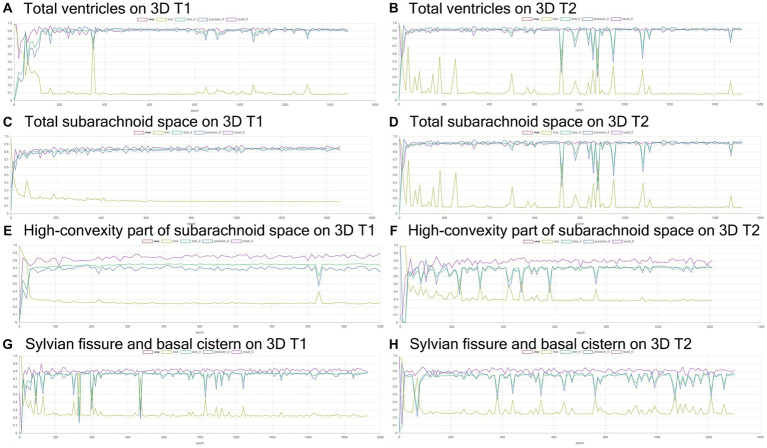
Inference results for internal validation of the semantic segmentation and image classification. The Dice scores (emerald green line), loss (lime green line), precision (blue), and recall (purple) for the automatically segmented volumes of the total ventricles **(A,B)**, total subarachnoid spaces **(C,D)**, high-convexity part of the subarachnoid space **(E,F)**, and Sylvian fissure and basal cistern **(G,H)** on 3D T1-weighted **(A,C,E,G)** and T2-weighted MRIs **(B,D,F,H)**.

**Figure 6:**
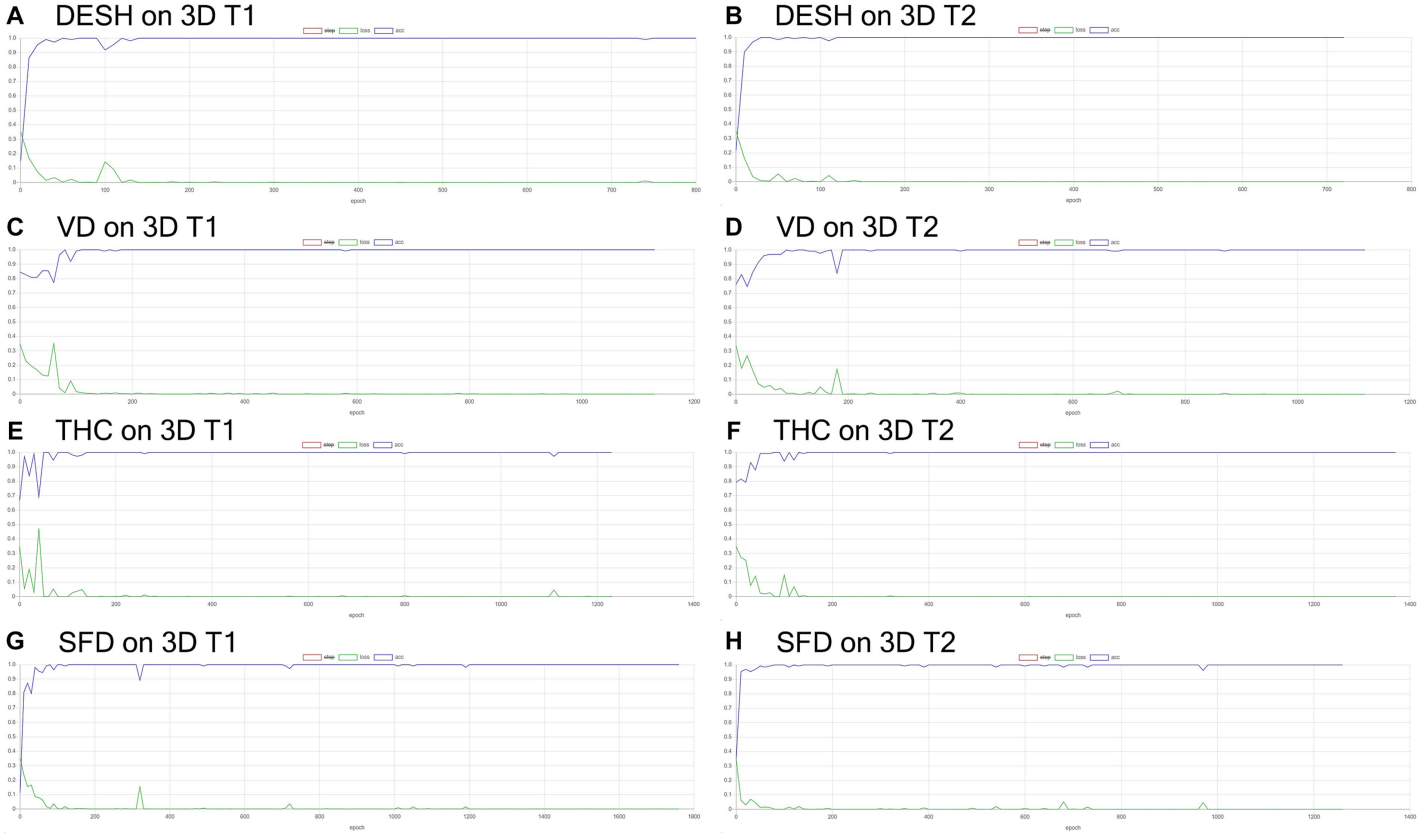
Results of training for deep learning of the image classification. The accuracy (blue line) and loss (green line) for the detection of disproportionately enlarged subarachnoid space hydrocephalus (DESH: **A,B**), ventricular dilatation (VD: **C,D**), tightened sulci in the high convexities (THC: **E,F**), and Sylvian fissure dilation (SFD: **G,H**) on 3D T1-weighted **(A,C,E,G)** and T2-weighted MRIs **(B,D,F,H)**.

**Figure 7:**
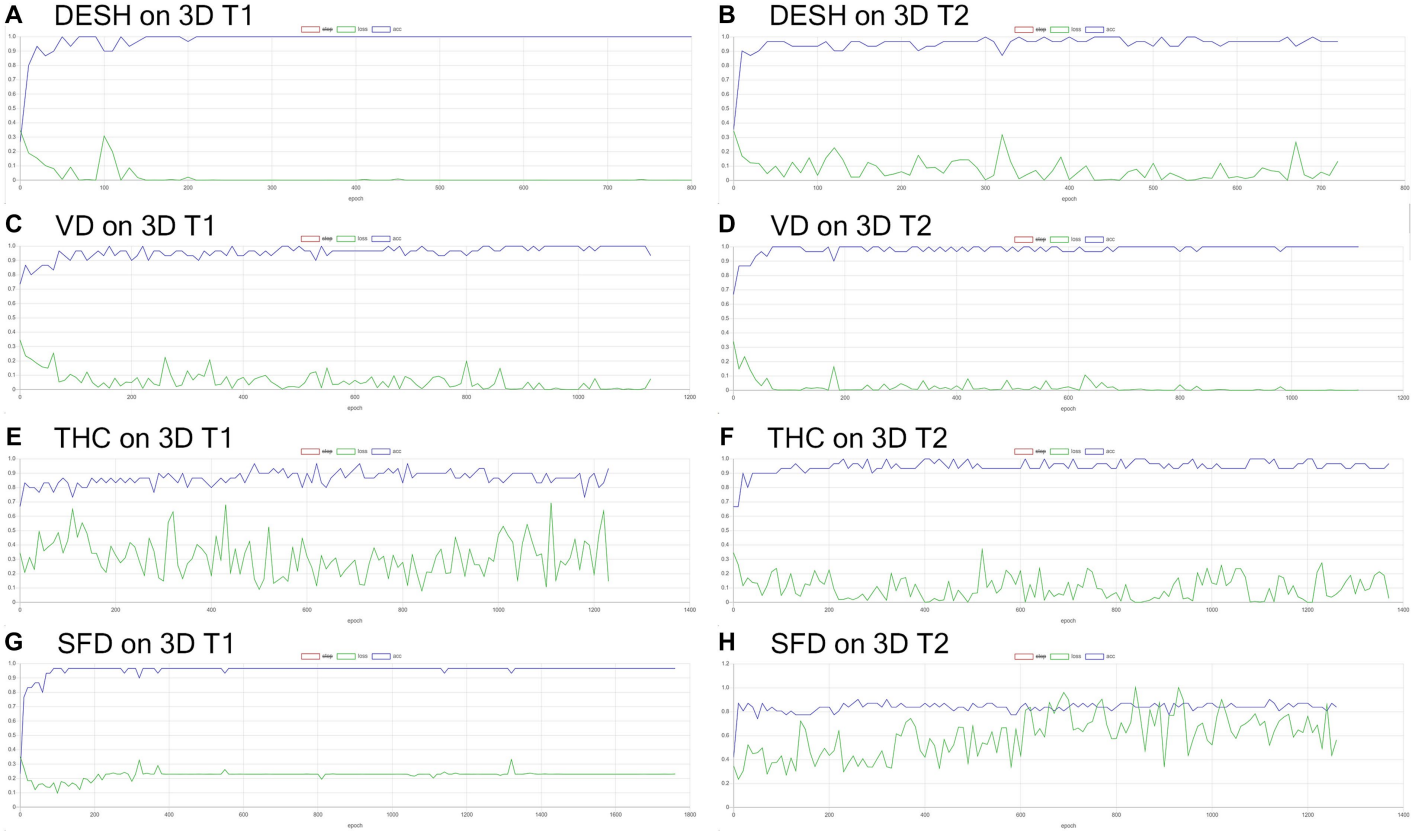
Inference results for internal validation of the image classification. The loss (green line) for the detection of disproportionately enlarged subarachnoid space hydrocephalus (DESH: **A,B**), ventricular dilatation (VD: **C,D**), tightened sulci in the high convexities (THC: **E,F**), and Sylvian fissure dilation (SFD: **G,H**) on 3D T1-weighted **(A,C,E,G)** and T2-weighted MRIs **(B,D,F,H)**.

**Figure 8:**
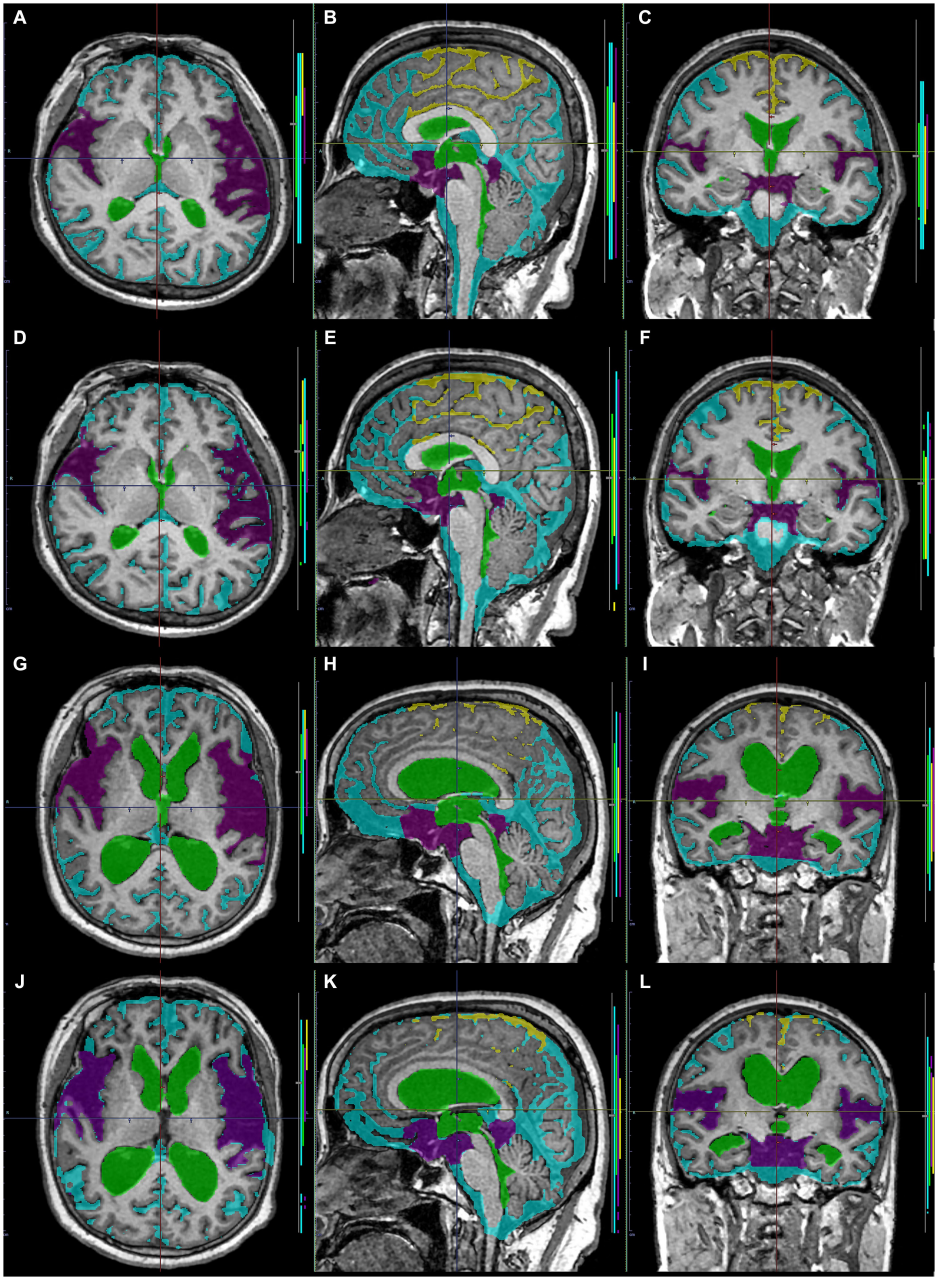
Comparison between manually and automatically segmented regions from 3D T1-weighted images. 3D T1-weighted images in a representative healthy volunteer **(A–F)** and a representative patient with Hakim disease and DESH **(G–L)**: manually segmented **(A–C,G–I)** and automatically segmented **(D–F,K–L)** volumes of the total ventricles (green); Sylvian fissure and basal cistern (purple); high-convexity part of the subarachnoid space (yellow); other subarachnoid spaces (marine blue) from 3D T1-weighted images.

**Figure 9:**
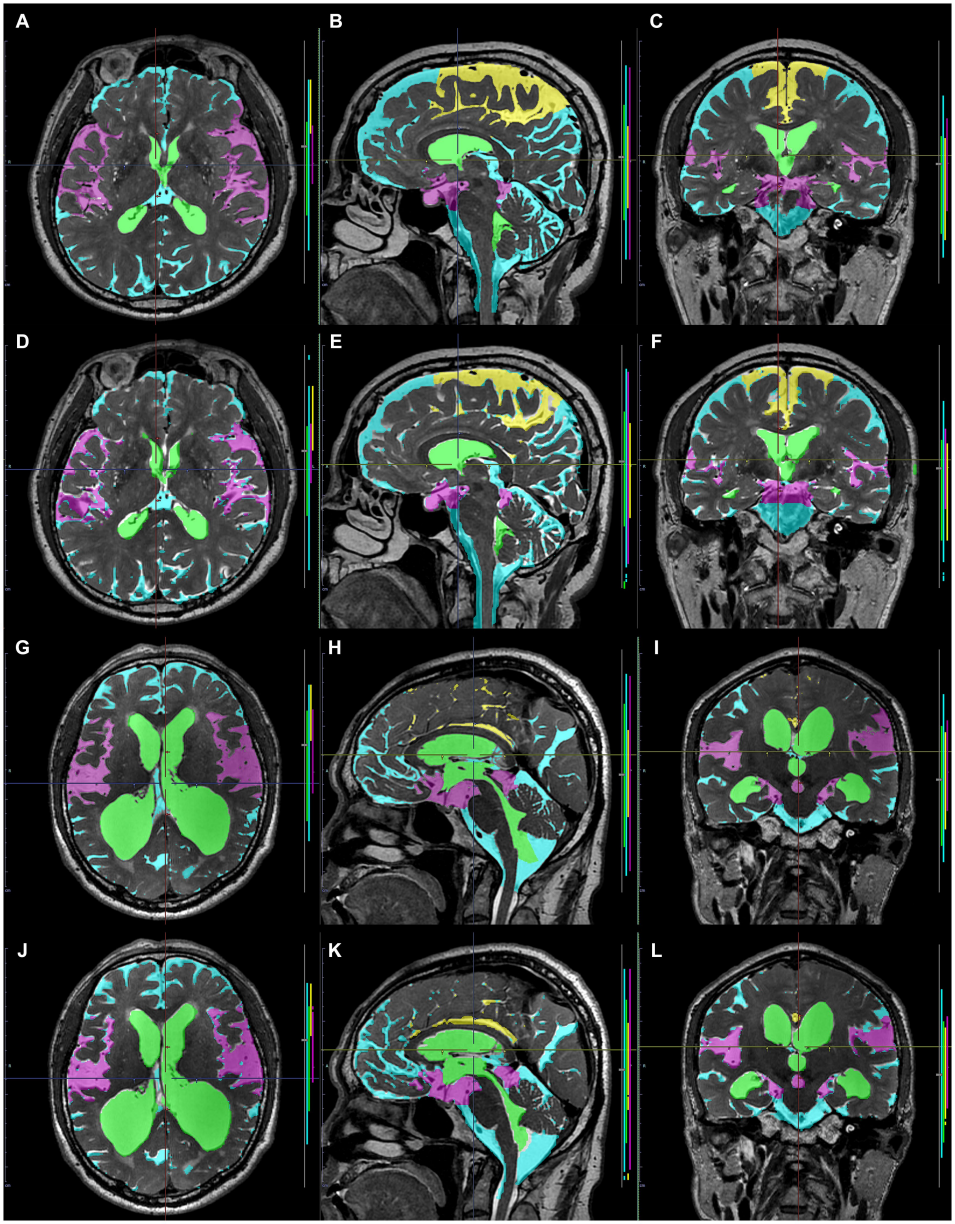
Comparison between manually and automatically segmented regions from 3D T2-weighted images. 3D T2-weighted images in a representative healthy volunteer **(A–F)** and a representative patient with Hakim disease and DESH **(G–L)**: manually segmented **(A–C,G–I)** and automatically segmented **(D–F,K–L)** volumes of the total ventricles (green); Sylvian fissure and basal cistern (purple); high-convexity part of the subarachnoid space (yellow); other subarachnoid spaces (marine blue) from 3D T2-weighted images.

Training and internal validation of the 3D U-Net model for semantic segmentation were repeated over 1,000 times (Figures 4–7; Supplementary Figures S1, S2). Overall, the intracranial CSF space, total ventricles, total SAS, Sylvian fissure and basal cistern, and the high-convexity SAS were segmented fully automatically from 3D T1-weighted (Figure 8) and T2-weighted MRIs (Figure 9). There was no significant difference between manually and automatically segmented volumes of the total ventricles, total SAS, high-convexity SAS, and Sylvian fissure and basal cistern (Table 3). Among the segmented regions, the mean Dice scores for the total ventricles were highest (0.85 from T1 and 0.83 from T2), those for the Sylvian fissure and basal cistern were second highest (0.70 and 0.69), and those for the high-convexity SAS were lowest (0.68 and 0.60). The mean Dice coefficient scores for all of the regions segmented from the T1-weighted image were superior to those from the T2-weighted image. The mean differences between the manually and automatically segmented volumes of the high-convexity SAS were smaller (T1 and T2; 3.6 mL and 4.2 mL) than those of the Sylvian fissure and basal cistern (5.3 mL and 8.3 mL).

**Table 3:** Comparison between mean (± standard deviation) automatically segmented and manually segmented volumes.

		Hakim disease		Normal					
	MRI	A-volume	M-volume	A-volume	M-volume	Dice score	*r*	*P*-A	*P*-M
Total ventricles	T1	129.2 ± 35.1	127.8 ± 35.5	26.9 ± 19.4	26.8 ± 18.9	0.85 ± 0.07	0.999	<0.001	<0.001
	T2	150.3 ± 45.2	150.1 ± 47.3	25.5 ± 9.9	25.2 ± 9.8	0.83 ± 0.06	0.997	<0.001	<0.001
Total subarachnoid space	T1	331.0 ± 35.7	329.9 ± 38.7	280.6 ± 60.6	287.4 ± 55.3	0.72 ± 0.04	0.980	<0.001	0.004
	T2	288.4 ± 53.2	298.5 ± 53.8	218.9 ± 57.7	247.3 ± 53.6	0.64 ± 0.06	0.867	<0.001	0.008
High-convexity part of subarachnoid space	T1	17.9 ± 3.7	21.5 ± 4.7	42.5 ± 12.1	42.2 ± 13.4	0.68 ± 0.07	0.900	<0.001	<0.001
	T2	7.3 ± 4.2	11.5 ± 5.2	32.2 ± 12.8	36.5 ± 11.0	0.60 ± 0.11	0.959	<0.001	<0.001
Sylvian fissure and basal cistern	T1	90.2 ± 12.0	95.5 ± 12.8	42.9 ± 10.3	45.6 ± 11.8	0.70 ± 0.05	0.969	<0.001	<0.001
	T2	88.6 ± 9.0	96.9 ± 17.9	37.7 ± 8.9	40.8 ± 9.9	0.69 ± 0.06	0.966	<0.001	<0.001

A-volume: automatically segmented region volumes with the 3D U-Net model. M-volume: manually segmented volume. Dice score; (overlapping 3D spaces) × 2/total area. The total area is defined as the sum of the ground truth image segmentation area (manually segmented) and the predicted area using the 3D U-Net model. r; Pearson’s correlation coefficient, to assess the correlations between volumes segmented manually and those segmented automatically by the 3D U-Net model. P-A; probability value of A-volume in Hakim disease vs. normal volunteer. P-M; probability value of M-volume in Hakim disease vs. normal volunteer.

### 3.3 Automatic quantitative assessment of DESH using image classification

**Figure 10:**
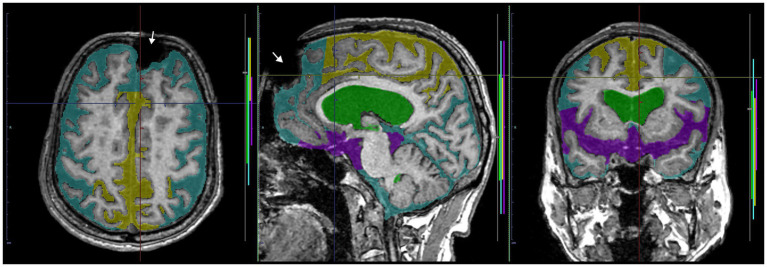
A case of discrepancy in DESH determination between AI and expert. MRI of an 84-year-old male volunteer, who claimed no specific history of head trauma showed a signal deficit (white arrow) in the left frontal region due to a metal artifact. The AI automatically judged the presence of DESH (softmax probability score: 1.0), VD (1.0), SFD (0.75), and the absence of THC (0.84), while the expert judged the presence of VD but not DESH, THC, or SFD. This case should have been excluded from the study.

The inference results of the presence or absence of DESH, VD, THC, and SFD with softmax probability scores are summarized in Table 4. All mean softmax probability scores were exceeded by 0.99, except for THC detection from the T1-weighted image (0.95) and SFD detection from the T2-weighted image (0.98). Among 99 images (49 T1 and 50 T2), only one T1-weighted image of a volunteer was judged by AI to have DESH, but the expert judged the subject to have no DESH (Figure 10). In addition, the discrepancy between the AI and expert determinations from T1-weighted MRIs was one for VD, one for THC, and three for SFD. However, AI determinations from T2-weighted MRIs were almost perfectly consistent with expert determinations, with only one discrepancy in VD determination. The accuracies for the determinations of DESH, VD, THC, and SFD by AI were 1.0, 1.0, 1.0, and 0.97 from T1-weighted MRIs, and 1.0, 1.0, 1.0, and 0.93 from T2-weighted MRIs, respectively.

**Table 4:** Softmax probability score for disproportionately enlarged subarachnoid-space hydrocephalus (DESH), ventricular dilatation (VD), tightened sulci in the high convexities (THC), and Sylvian fissure dilatation (SFD).

	3D T1-WI MRI			3D T2-WI MRI		
Total number	49			50		
Male: female	24: 25			25: 25		
Hakim disease: normal	12: 37			15: 35		
	Expert	AI	Probability score	Expert	AI	Probability score
DESH	12	13	0.997 ± 0.020	15	15	0.991 ± 0.059
VD	14	13	0.999 ± 0.006	14	15	0.991 ± 0.060
THC	12	11	0.946 ± 0.121	15	15	1.000 ± 0.002
SFD	9	12	0.992 ± 0.039	12	12	0.981 ± 0.060

Expert; two experts in the imaging diagnosis of Hakim disease reached judgments through consensus reading. AI; judgments were automatically assessed using the multimodal CNN.

### 3.4 DESH detection from 3D volumetric indices of automatically segmented regions

All DESH, Venthi and Sylhi indices, calculated by the manually and automatically segmented volumes on T1-weighted and T2-weighted MRIs, had sufficiently high AUCs (>0.996), specificities (>0.944), and sensitivities (>0.923) (Table 5). However, optimal thresholds calculated to maximize the sum of sensitivities and specificities for detecting DESH differed between manually and automatically segmented volumes and between T1-weighted and T2-weighted MRIs.

**Table 5:** Area under the receiver-operating characteristic curve (AUC) and optimal thresholds with 95% confidential interval (CI) for detecting DESH.

	MRI	AUC (95% CI)	Specificity (95% CI)	Sensitivity (95% CI)	Optimal threshold
Automatically segmented region volumes with the 3D U-Net model					
DESH index	T1	0.996 (0.986–1.00)	0.944 (0.861–1.00)	1.00 (1.00)	2.563
DESH index	T2	1.00 (1.00)	1.00 (1.00)	1.00 (1.00)	7.578
Venthi index	T1	1.00 (1.00)	1.00 (1.00)	1.00 (1.00)	1.573
Venthi index	T2	1.00 (1.00)	1.00 (1.00)	1.00 (1.00)	3.370
Sylhi index	T1	0.966 (0.898–1.00)	1.00 (1.00)	0.923 (0.769–1.00)	3.083
Sylhi index	T2	1.00 (1.00)	1.00 (1.00)	1.00 (1.00)	4.046
Manually segmented volumes					
DESH index	T1	1.00 (1.00)	1.00 (1.00)	1.00 (1.00)	3.980
DESH index	T2	1.00 (1.00)	1.00 (1.00)	1.00 (1.00)	5.678
Venthi index	T1	0.999 (0.986–1.00)	0.992 (0.977–1.00)	1.00 (1.00)	1.681
Venthi index	T2	1.00 (1.00)	1.00 (1.00)	1.00 (1.00)	2.760
Sylhi index	T1	1.00 (0.999–1.00)	0.977 (0.992–1.00)	1.00 (1.00)	2.157
Sylhi index	T2	1.00 (1.00)	1.00 (1.00)	1.00 (1.00)	2.771

DESH index: total ventricular volume and Sylvian fissure and basal cistern volume divided by the high-convexity part of the subarachnoid space volume. Venthi index: Ventricular volume divided by the high-convexity part of the subarachnoid space volume. Sylhi index: Sylvian fissure and basal cistern volume divided by the high-convexity part of the subarachnoid space volume.

## 4 Discussion

In this study, we developed an automatic quantitative assessment of DESH from 3D T1-weighted or T2-weighted MRIs, supplementally measuring segmented volumes and indices related to DESH by using two combined AI models: a 3D U-Net for semantic segmentation, and a multimodal CNN for image classification. A previous study on automatic region extraction from 3D MRI using AI in hydrocephalus have involved the automated extraction of ventricles, subarachnoid space, and intracranial CSF space (Grimm et al., 2020). However, there have been no previous reports on AI-based detection of DESH, including VD, THC, and SFD, from these automatically extracted regions. Currently, DESH, VD, THC, and SFD are evaluated subjectively and evaluators often differ in judgment (Sasaki et al., 2008; Ishikawa et al., 2010; Narita et al., 2016; Shinoda et al., 2017). This ambiguity is often influenced by a patient’s background, e.g., living and family environment or co-morbidities. Although the typical Hakim patient presents with the triad of cognitive decline, gait and balance impairment, and urinary incontinence, there are actually many Hakim patients who have only cognitive decline or only gait and balance impairment (Yamada et al., 2017a, 2021a, Nakajima et al., 2021). Many of these patients might not be diagnosed with Hakim disease due to overlooked DESH on CT scan or MRI, and are often misdiagnosed for years as having Alzheimer’s disease (Yamada et al., 2016b; Irie et al., 2020; Nakajima et al., 2021; Luca et al., 2023) or Parkinson’s disease (Stolze et al., 2001; Picascia et al., 2019; Mostile et al., 2023) leading to progression of these symptoms. Therefore, the AI-based decision support tool can be expected to give patients with Hakim disease a better chance of receiving appropriate treatment earlier, to reduce ambiguity in the interpretation of DESH, and to decrease potential anchoring bias. Quantitative measurements and indices ensure objectivity and allow for easier interpretation of classification results, especially in cases where the clinical diagnosis is not clear.

This study has some limitations. First, for training and validation datasets, the predefined subregions of the ventricles, high-convexity SAS, and Sylvian fissure and basal cistern were manually segmented. In our previous reports (Yamada et al., 2015; Yamada and Mase, 2023), however, the reproducibility and validity of our 3D manual segmentation method were verified. Second, domain shift, differences in imaging among facilities that lower performance, is a common but critical issue in AI based segmentation and detection (Takahashi et al., 2021). Therefore, our deep learning models used two different sequences of 3D T2 Cube and SPACE on three different MRI equipment devices (GE Healthcare and Siemens AG). Third, the control group in this study was significantly younger than the patient group. To address this issue, we used covariates (such as age and gender) as input to the multimodal CNN model.

For future perspectives, we plan to develop a new app based on the results of this study in the near future. In addition, using this app, we are prepared to conduct the next study to validate its accuracy and determine appropriate cutoff values for the segmented regions and DESH, Venthi, and Sylhi indices in other large cohorts, including elderly community-dwelling populations and Hakim patients with or without Alzheimer’s disease.

## 5 Conclusion

Our combined deep learning models could automatically detect DESH, which is the key imaging marker for Hakim disease (iNPH) from 3D T1- or T2-weighted MRI with automatically segmented volumes. The results of the AI-based segmentation seemed to outperform the manual segmentation by experts. Our AI-based diagnostic imaging support with quantitative assessment of DESH might contribute to improved diagnostic accuracy of Hakim disease (iNPH), might certainly reduce the number of missed and misdiagnosed Hakim disease (iNPH), and could be applied in future multicenter collaborative studies. The social implementation of AI-based diagnostic imaging support systems and medical software is advancing rapidly, but regulatory and ethical aspects need to be carefully considered.

## Data availability statement

The datasets presented in this article are not readily available because the MRI data in this study is not available to the community via any open repositories, because the ethics committees have approved the sharing of the MRI data in this research with collaborative institutes and does not allow its being provided to other institutions. The data will be available only on the condition that the ethics committees approve any new participation in the collaborative research. Requests to access the datasets should be directed to SY, shigekiyamada393@gmail.com.

## Ethics statement

The studies involving humans were approved by the study design and protocol have been approved by the ethics committee at Shiga University of Medical Science on October 11, 2019 (IRB number: R2019-227) and by the ethics committee at Nagoya City University Graduate School of Medical Science on December 1, 2022 (IRB number: 60-22-0083). The studies were conducted in accordance with the local legislation and institutional requirements. The participants provided their written informed consent to participate in this study.

## Author contributions

SY: Conceptualization, Data curation, Formal analysis, Funding acquisition, Investigation, Methodology, Project administration, Resources, Software, Validation, Visualization, Writing – original draft. HI: Investigation, Methodology, Software, Validation, Writing – review & editing. HM: Methodology, Software, Validation, Writing – review & editing. SI: Supervision, Writing – review & editing. TO: Supervision, Writing – review & editing. MT: Supervision, Writing – review & editing. CI: Conceptualization, Supervision, Writing – review & editing. YW: Funding acquisition, Supervision, Writing – review & editing. SW: Supervision, Writing – review & editing. MO: Funding acquisition, Supervision, Writing – review & editing. MM: Supervision, Writing – review & editing.
