# Supplementary figures and images for: Automatic assessment of disproportionately enlarged subarachnoid-space hydrocephalus from 3D MRI using two deep learning models
Source: Front Aging Neurosci. 2024 Mar 15;16:1362637. doi: 10.3389/fnagi.2024.1362637 (PMC10978765; doi:10.3389/fnagi.2024.1362637)

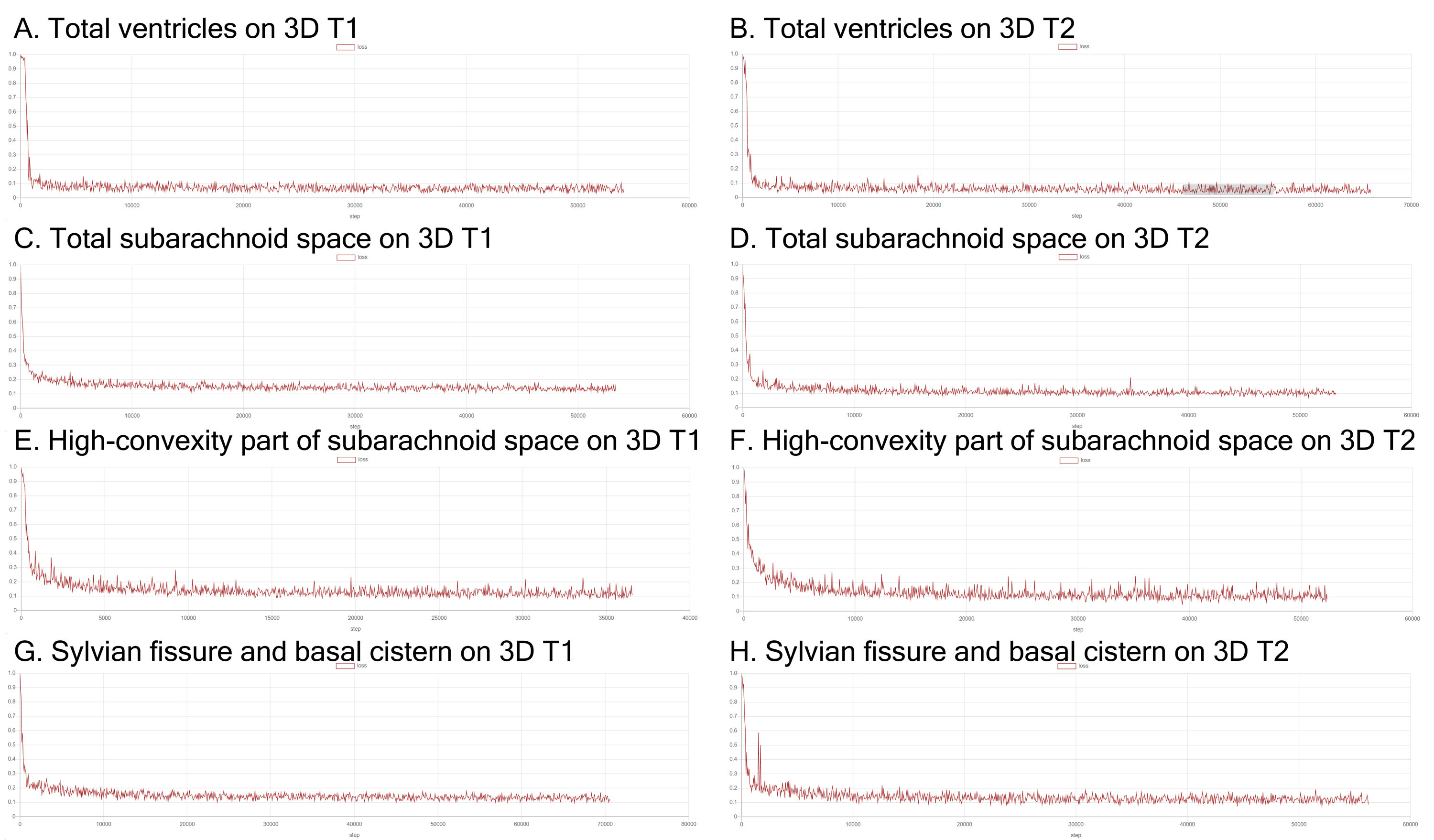

Supplement: Supplementary file 5 [file Image_1.JPEG]

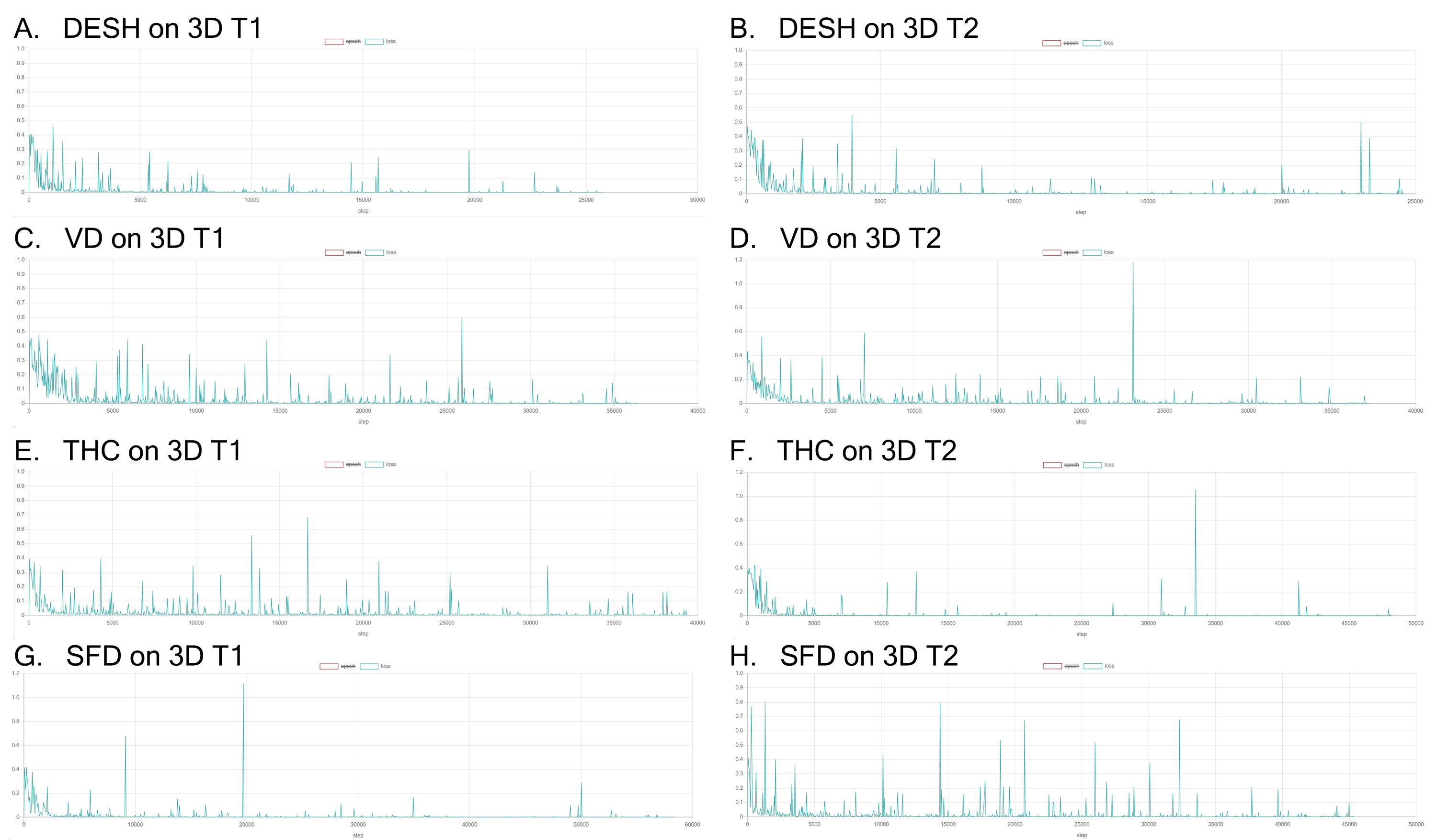

Supplement: Supplementary file 6 [file Image_2.JPEG]
